# Restoration of Endodontically Treated Teeth Review and Treatment Recommendations

**DOI:** 10.1155/2009/150251

**Published:** 2010-01-26

**Authors:** Iris Slutzky-Goldberg, Hagay Slutzky, Colin Gorfil, Ami Smidt

**Affiliations:** ^1^Post-Graduate Program in Endodntics, HUHSDM, Jerusalem 91120, Israel; ^2^Department of Community Dentistry, HUHSDM, Jerusalem 91120, Israel; ^3^Department of Prosthodontics, The Maurice and Gabriella School of Dental Medicine, TAU, Tel Aviv 69978, Israel; ^4^The Center for Graduate Studies in Prosthodontics, HUHSDM, Jerusalem 91120, Israel

## Abstract

Coronal restorations and posts can positively influence the long-term prognosis of teeth following root canal therapy. Final sealing the canal by placing an appropriate post and core will minimize leakage of oral fluids and bacteria into the periradicular area and is recommended as soon as possible after completion of root canal filling. Glass ionomer or MTA placed over the residual root canal filling after post space preparation may be effective to prevent bacterial leakage. A ferrule of 1-2 mm of tooth tissue coronal to the finish line of the crown significantly improves the fracture resistance of the tooth and is more important than the type of the material the core and post are made of.

## 1. Introduction

Recently, growing attention has been given to procedures carried out after completion of the endodontic treatment and their impact on the prognosis of devitalized teeth. These procedures may allow the passage of microorganisms and their by-products to the apical region of the root and into the alveolar bone, a potential cause of delayed failures. The consequences of these “events” may be important in determining the long-term success of the endodontic treatment [1].

Ray and Trope [2] evaluated the relationship between the quality of the coronal restoration and the quality of the root canal filling by examining the radiographs of endodontically treated teeth. They observed that a combination of good restorations and good endodontic treatments resulted in absence of periapical inflammation in 91.4% of the teeth, whereas poor restorations and poor endodontic treatments resulted in the absence of periradicular inflammation in only 18.1% of the teeth examined. Furthermore, where poor endodontic treatments were followed by good permanent restorations, that appeared radiographically sealed, the resultant success rate was 67.6%. They concluded that apical periodontal health depended significantly more on the coronal restoration than on the technical quality of the endodontic treatment. The importance of a good restoration to the periapical health was confirmed in similar studies [3–6], even though these demonstrated that an adequate root filling had a more substantial impact on the outcome of treatment than the quality of the coronal restoration [7].

## 2. Root Canal Filling Materials

Salivary microleakage is considered to be a major cause of endodontic failure due to bacteria and endotoxins penetration along the root canal filling [8, 9]. Contamination of the root canal can occur through salivary microleakage during post space preparation, after post cementation, through temporary fillings, and through leaking margins of permanent restorations [1].

**Figure 1:**
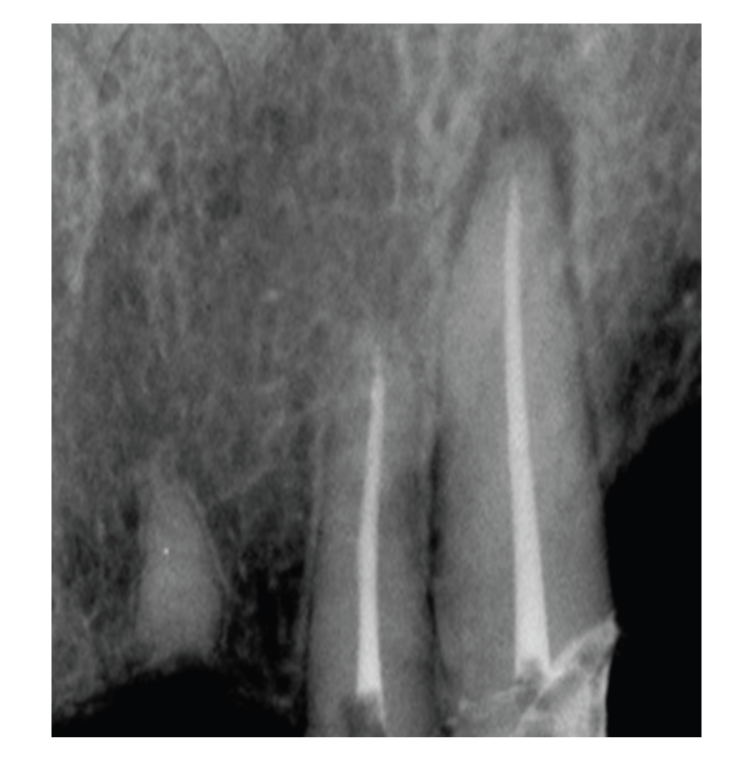
Bacterial contamination occurred after completion of root canal treatment in the tooth, which remained with a temporary filling for 15 month.

The penetration of saliva through obturated root canals increases with the longer exposure period [10] (see Figure 1). Microorganisms were isolated from obturated root canals after 22 days of exposure to saliva. Both lateral and vertical condensations methods of obturation were evaluated in this study [11]. Additionally leakage of obligate anaerobes and bacterial metabolites along laterally condensed root canals was demonstrated without any significant differences between root canals obturated with gutta-perch cones (GP) and other root canal sealers [12–14].

A novel filling system that was introduced in 2004, Resilon and Epiphany, was no better than gutta-percha with Roth or with epoxy resin sealers like AH Plus or MM-seal at sealing root canals [15]. In a comparative study using a microleakage model and a new sequence detection assay “One Cut Event AmplificatioN (OCEAN) technique”, Pasqualini et al. [16] demonstrated that canals obturated with Resilon showed a greater number of microleakage events than those obturated with gutta-percha and Zinc oxide eugenol sealer. On the other hand, Bodromlu et al. [17] showed better results for Resilon as compared with gutta-percha and AH-Plus, especially in delayed post space preparation. Although none of the root-canal filling materials and sealers exhibited complete apical sealing [18]. In another study, the Resilon system provided the lowest mean values of apical leakage, but did not provide hermetic sealing of the root canal system, furthermore, thermoplastification negatively influenced the apical sealing ability of Resilon [19].

## 3. Temporary Filling Materials

Temporary fillings, in teeth undergoing root canal treatment or before completion of the final restoration, must provide an effective barrier against salivary contamination of the root canal. Intermediate Restorative Material (IRM), Cavit, and TERM are commonly used as temporary filling materials [20]. IRM, that is used due to its high compressive strength [21], has been demonstrated in bacterial leakage to be less leak proof than Cavit and TERM [20, 22]. These results were similar to those reported by others, in experiments performed using the fluid filtration technique [23, 24] and the dye penetration technique [25].

Some authors speculate that the expansion of hygroscopic restorative materials leads to poor adaptation at the restorative material-cavity walls interface [26, 27]. In an in vitro work which examined the antibacterial effect of 4 temporary filling materials, IRM had a bacteriocidic effect on the growth of S. mutans which lasted for at least 14 days, whereas it had a bacteriostatic influence for 1 day on the growth of E. faecalis, which could not be demonstrated after 7 days. The authors suggested that IRM may be selective for some bacteria but not for others, therefore allowing the growth of E. faecalis which is associated with failure of endodontic treatments [28].

## 4. Are Endodontically Treated Teeth More Brittle?

Contrary to common belief, endodontically treated teeth are not more brittle [29, 30]. No difference in moisture content was found between endodontically treated and vital teeth [31]. The access cavity in combination with an early loss of one or both marginal ridges leaves the tooth at serious risk. According to Dietschi et al. [32], the loss of vitality and a proper RCT affected tooth biomechanical behavior only to a limited extent. The tooth strength is reduced in proportion to coronal tissue lost, due to either carious lesions or restorative procedures. A direct relationship exists between the amount of remaining tooth structure and the ability to resist occlusal forces [33] (see Figure 4). It is therefore important to provide a restoration allowing cuspal coverage as soon as possible after completion of the RCT [1].

Anterior teeth with minimal access cavity can be restored with a composite resin, and premolars and molars with minimal access cavities or other coronal tissue loss can be restored with amalgam or composite resin in combination with a resin bonding system. Whereas, posterior teeth with large access cavities following extensive carious lesions carry greater occlusal loads and therefore require protection against possible fracture [34, 35]. Notwithstanding, the use of posts does not reinforce endodontically treated teeth and some reports even show that teeth which were restored without a post and core are less susceptible to fracture than teeth with post and core [36].

Several materials are available for core buildup: gold, amalgam, resin composites, and reinforced glass ionomers [36]. In a study that compared the use of amalgam, resin composite, and glass ionomer in combination with a prefabricated post in extracted teeth subjected to masticatory forces, it was found that amalgam had the lowest failure rate, and that glass ionomer core buildup materials caused the greatest number of failures [37]. Some studies supported the use of amalgam dowels in the root canals to increase the retention of the restoration when the remaining wall thickness was less than 4 mm [38, 39]. Nonetheless, Tamse et al. [40] compared 49 mesial roots extracted due to vertical fractures with 52 mesial mandibular roots without fractures, and found that 67.3% of the vertically fractured roots had an amalgam dowel in the coronal part (1-2 mm) of the root. The authors suggested that the removal of more dentin from the coronal part of a susceptible to fracture root and condensing amalgam with an expansion potential into an already weakened root contributes to vertical fracture development.

In an in vitro study by Pilo et al. [41], comparing resin-based composite, amalgam, and cast gold as core materials under a crown in endodontically treated teeth, no significant difference in fracture and failure characteristics was found among these materials, provided a 2-mm ferrule existed on the margin of healthy tooth substance. These findings are in accordance with Hoag and Dwyer [42] who suggested that 1-2 mm of tooth tissue coronal to the finish line of the crown significantly improves the fracture resistance of the tooth. The ferrule reduced vertical root fracture by one third, and when failure occurred, it is usually in the form of horizontal root fracture—thus, leaving the teeth more likely to be retrievable. Nonetheless, following enlargement of the cavity size, especially after endodontic access, the tooth is subjected to an increased cuspal deflection and a potential fracture [43].

A post should be used only when there is insufficient tooth substance remaining to support the final restoration [44]. Post length should be as long as the clinical crown height. The post should end halfway between the crestal bone and the root apex [45]. Short posts have poor retention and transmit larger lateral forces to the remaining root structure [46]. It was also suggested that increasing post length is more important than post diameter for retention improvement [47].

## 5. Microleakage after Post Space Preparation

During post space preparation a small volume of obturation material remains in the root canal. This residual filling at the apical region serves as the last barrier against microbial penetration along the root canal, which may in time cause periapical inflammation. The consequences of these events are contamination of the canal and colonization of bacterial species at the walls of the apical portion of the root canal [48–50]. The length of GP fill remaining in the root canal has a major effect on the apical seal. There is a consensus that a longer filling provides a better seal [51, 52]. It was observed that when only the apical 4 mm of root canal filling (RCF) remained, the leakage was significantly greater than when the original full-length filling was examined [53]. DeCleen [51] stated that 3 mm of remaining GP is the absolute minimum and that preferably 6 mm should be left in the root canal.

The methods of canal obturation, post space preparation, and timing of post space preparation may influence future microleakage. Haddix et al. [54] compared heated pluggers, gates-glidden drills, or GPX instruments as post space preparation tools. The remaining length of apical GP fillings was 3 or 5 mm. At these levels, significantly less leakage was observed when the heated plugger technique was used. This may be explained by the additional effect of vertical condensation achieved by using heated pluggers [1]. Using a fluid transport device, Fan et al. [55] found more leakage after delayed post preparation than after immediate preparation. The root canals were obturated with laterally condensed GP cones and either AH26 or Pulp Canal Sealer, noting no significant difference between the sealers. Wu et al. [53] suggested that leakage following removal of the coronal portion of the RCF during post space preparation may be compensated for by the cemented post. In an SEM analysis of the canal walls after post space preparation, large areas were covered by smear layer, debris, and sealer/gutta-percha remnants. Thus, the post space may not be available for adhesive bonding and resin cementation of fiber posts [56].

## 6. Post Cementation

The influence of the gap between the post and the residual gutta-percha on the clinical outcome of endodontically treated teeth was studied from records of patients treated by dental students. 3 groups of teeth were compared according to the gap between the post and the residual gutta-percha. The best outcome was found in teeth where the post was in contact with the gutta-percha (83.3%); when this gap was 0–2 mm a satisfactory outcome was found in 53.6% of the teeth, and a gap larger than 2 mm resulted in an inferior outcome, where only 29.4% of the teeth were defined satisfactory [57] (see Figure 3). A fluid filtration system was used to examine the effect of cementation of stainless-steel post and a carbon-fiber post system on microleakage. Both posts, when cemented with dentin-bonding resin cements (C & B Metabond and Panavia-21), exhibited less microleakage than when the posts were cemented with non-dentin-bonding cements (glass ionomer and zinc phosphate) [58]. It was demonstrated that significantly greater leakage occurred in temporary restorations than in cast post and cores cemented with zinc phosphate cement or prefabricated posts and cores cemented with a composite luting cement [59]. These results were advocated by another study which demonstrated, in addition, that the use of dentin-bonding cements resulted in less microleakage than with traditional, non-dentin-bonding cements, and that the adaptation of the post to the canal may be more important than the cement used [60].

Fogel [61] compared microleakage of five post systems using the fluid filtration system: stainless steel posts cemented with (a) zinc phosphate cement, (b) polycarboxylate cement, (c) a composite resin, (d) composite resin after use of a dentin bonding agent, and (e) composite resin after use of a dentin conditioner and a dentin bonding agent. The results showed that none of the post-cement systems tested were capable of consistently achieving a fluid-tight seal. Usumez et al. [62] compared the sealability of stainless steel dowels (ParaPost), (2) glass fiber dowels (Snowpost), (3) resin-supported polyethylene fiber (Ribbond) dowels, or (4) zirconia dowels (Cosmopost). Resin-supported polyethylene fiber dowels and glass fiber dowels tested exhibited less microleakage compared to zirconia dowel systems. The use of various types of fiber-reinforced posts and resin cement is becoming more popular. Among different types of adhesively-luted fiber-reinforced dowels evaluated: DT Light Post (LP), Glassix (GL), Ribbond (RB), and StickTech Post (ST), the individually shaped polyethylene-reinforced dowel (Ribbond) showed the least overall leakage [63]. In microleakage study of 200 endodontically treated teeth restored with prefabricated dowels and tooth-colored restoratives as core materials with and without the use of a flowable composite liner, the use of flowable liners reduced microleakage. Z-100 both with and without flowable liner demonstrated better resistance to leakage as compared with Solitaire, Admira, and Filtek P60 [64].

A recent systematic review of post and core materials conducted by Theodosopoulou and Chochlidakis [65] was based on articles found in an electronic search of MEDLINE from 1966 to 2008, and a Cochrane and EMBASE search from 1945 to 2008. It was aimed to determine which post and core system is the most successful when used in vivo to restore endodontically treated teeth. Failures were considered as cases with root fracture, dowel fracture, periapical radiographic change/lesion, and/or dowel dislodgment. The following conclusions were made: Carbon fiber in resin matrix dowels (Composiposts) are significantly better than precious alloy cast dowels. Glass fiber dowels are significantly better than metal screw dowels, and moderately better than quartz fiber dowels. and glass fiber reinforced dowels (Postec) are better than quartz fiber dowels (AEsthetiplus). Glass fiber-reinforced dowels are moderately worse than titanium dowels. Furthermore, quartz fiber dowels (DT) and glass fiber-reinforced dowels (Postec) show the same results when compared to each other.

## 7. Permanent Restoration

Friedman and Mor [66] stated that endodontic treatment is a predictable procedure with long-term tooth retention rate, and that asymptomatic teeth, in spite of having a periapical lesion, may be considered functional. Indeed, Salehrabi and Rotstein [67] checked the records following initial root canal treatments of 1,462,936 teeth from 1,126,288 patients and found that 97% of the teeth remained in the oral cavity after an evaluation period of 8 years. The 3% remaining were subjected to apical surgeries, extractions, and so forth, most of which occurred within 3 years from completion of treatment. Complications occurred in teeth without any coronal coverage in 85% of the cases. In another study, endodontically treated teeth not crowned after obturation were lost 6 times more often than teeth crowned after obturation [68]. A 10-year prospective clinical trial, showed 94% survival rate of metal post-and-cores with a crown [69]. Another 17-year controlled prospective study showed that the type of core restorations under the crowns had no effect on the survival rate of 307 endodontically treated teeth [70]. This was confirmed in another study, which showed that the type of post and core was not relevant with respect to survival. However, the longevity of a post-and-core restoration was influenced by the amount of remaining dentin height after preparation [71].

## 8. Timing

The dilemma of whether to place a permanent restoration immediately after completion of the endodontic treatment or to wait for the resolution of the rarefying osteitis exists among dental practitioners. Safavi et al. [72] examined the influence of delaying coronal permanent restorations on the prognosis of endodontically treated teeth, 464 endodontically treated teeth were evaluated, using follow up radiographs. Higher success rate was found in teeth with permanent restorations (amalgam, composite resin filling, or cast crowns with or without a post and core) than in teeth with temporary fillings (IRM or Cavit).

Although the difference was not significant, they suggested that an appropriate and prompt permanent restoration after completion of endodontic treatment should be carried out. It showed significantly more leakage after placing a temporary filling than following placement of a permanent restorative material to seal access cavities [26]. The authors suggested that it may be more prudent to use permanent restorative materials for provisional restorations in order to prevent inadequate canal sealing and the resulting risk of fluid penetration. Another study compared the amount of dye leakage after post space preparation along root canals obturated with GP with either AH26 or a zinc oxide eugenol (ZOE) based sealer. The post space was prepared immediately after obturation or one week later. The only significant difference was that in the delayed preparation group in which ZOE based sealer was used; there was greater leakage [73].

## 9. Coronal Barrier

Metzger et al. [74] compared remaining gutta-percha after post space preparation of 3, 5, 7, or 9 mm and found that the seal of 3, 5, and 7 mm remaining Gutta-percha was inferior to an intact filling of 14 mm. Microbial leakage of E. faecalis after post space preparation in teeth filled in vivo with RealSeal versus Gutta-percha was examined in teeth in which 5 mm of apical filling material was left. RealSeal-filled teeth showed leakage after 3.5 days and Gutta-percha-filled teeth showed a mean leakage of 10 days [75].

Since the gutta-percha remaining after post space preparation does not provide a seal equivalent to the intact root canal filling [1], several materials and techniques have been suggested to address the shortcomings of gutta-percha. Numerous studies have shown that the use of intraorifice barriers in canals filled with gutta-percha significantly decreases coronal microleakage [76–78]. Yamauchi et al. [79] demonstrated substantial reduction in apical periodontitis in dogs' teeth after placement of a coronal barrier using IRM or dentin bonding/composite resin. The differences in the inflammation rate between the group without plugs (89%) and those with IRM (38%) or composite orifice plug (39%) were statistically significant. In their study even when only gutta-percha (without sealer) was used for filling, the placement of an orifice plug significantly prevented the occurrence of inflammation. The results of this study demonstrate that the placement of an orifice plug after root canal filling is beneficial to delay and prevent coronal microleakage. It may be assumed that the placement of an intraorifice barrier establishes an immediate coronal seal.

Outcomes of studies underscore the importance of a sound coronal seal with respect to the overall success of root canal treatment. Pisano et al. [78] tested whether Cavit, IRM, or Super-EBA as intraorifice filling materials can prevent coronal microleakage of human saliva and its components in the absence of a coronal restoration. At the end of a 90-day test period, 15% of the Cavit-filled orifices leaked, whereas 35% of the IRM and Super-EBA-filled orifices leaked.

The gutta-percha obturated root canals that received an intraorifice filling material leaked significantly less than the obturated unsealed control group, all of which leaked in less than 49 days. Another study evaluated the effect of glass ionomer used as an intracoronal barrier for the prevention of microleakage by using the fluid transport model. Following root canal treatment 30 teeth received 0–2 mm intracoronal barrier of “Triage” glass ionomer. 1 or 2 mm of Triage significantly reduced coronal microleakage in thermocycled endodontically treated teeth as compared with no barrier [80].

A study which compared coronal microleakage between Resilon with Epiphany primer and sealer and gutta-percha with 2-mm of Triage intraorifice barrier using a fluid filtration model found significantly less leakage for the gutta-percha/glass-ionomer intraorifice barrier group than the Resilon alone group [81].

Mineral trioxide aggregate (MTA) was also suggested for use as a coronal barrier, in a study that compared the effectiveness of grey MTA, white MTA, and Fuji II LC cement as coronal barriers to bacterial leakage. A dual chamber leakage model utilizing salivary microbes was used for the evaluation of the microleakage. Leakage did not occur until day 52 with Fuji II, day 56 with gray MTA, and day 59 with white MTA. There was no statistically significant difference in leakage between the materials tested at 30, 60, or 90 days [82]. Another study showed that after 10 months, there were no demonstrable differences between periapical inflammation in dog teeth with conventional root fillings and those coronally augmented by MTA [83]. Mavec et al. [84] suggested that in clinical situations of teeth with compromised crown-root ratio that require a post and core, 1 mm of Vitrebond over 2 or 3 mm remaining gutta-percha could reduce the risk of recontamination of the apical gutta-percha.

## 10. Discussion and Recommendations

Bacteria harbored the oral cavity can cause periapical inflammation by penetrating the root canal not only before or during the endodontic treatment but also during the prosthetic treatment or after its completion. The need for an immediate and proper restoration after endodontic treatment is of utmost importance [1]. An appropriate and prompt restoration of the tooth after completion of endodontic treatment is highly recommended [50, 72]. Failing to place a permanent restoration may result in a higher rate of tooth loss [68–70]. If a permanent restoration cannot be completed at the end of the endodontic treatment, it was suggested first to place Cavit or a similar material with good sealing abilities and then IRM for its high compressive strength [25].

**Figure 2:**
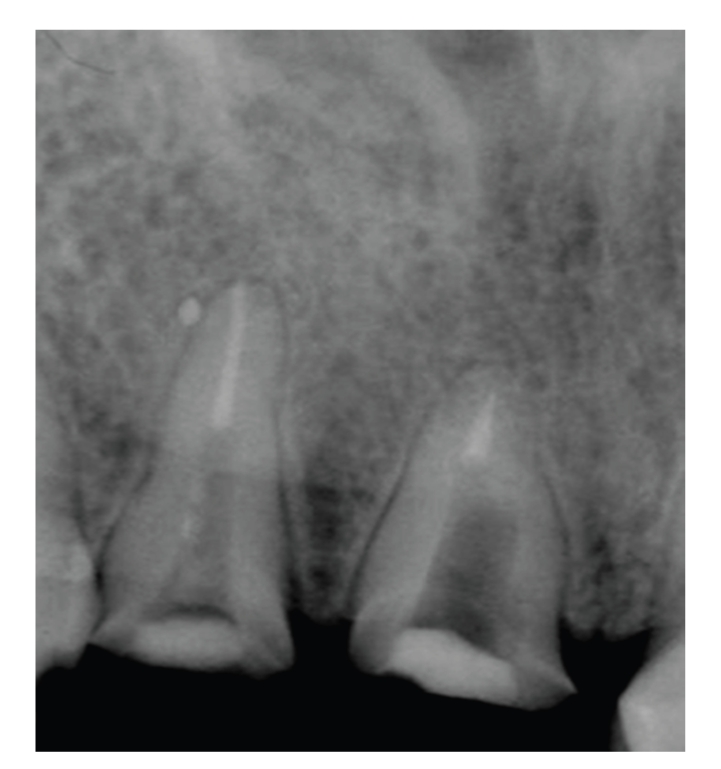
The post space should be dressed between appointments and irrigated before post cementation.

**Figure 3:**
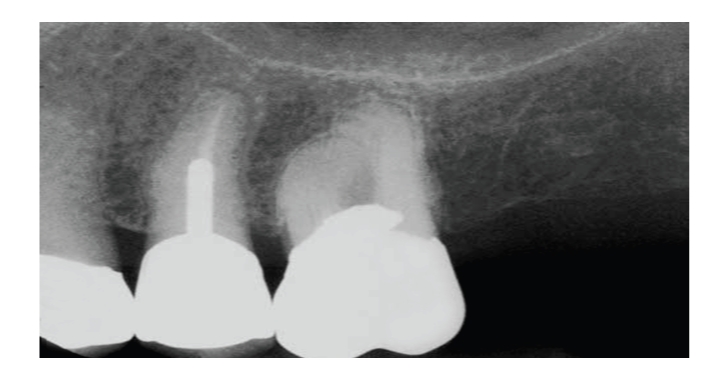
Best outcome found in teeth where the post was in contact with the gutta percha.

**Figure 4:**
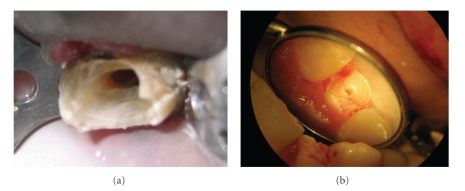
The access cavity in combination with an early loss of both marginal ridges due to caries (a) or trauma (b) leaves the tooth at serious risk of fracture.

When 2 or more walls are missing, an addition of a post is required to restore the tooth [36]. The space prepared for a cast post should be regarded as an unsealed root canal. It may therefore become contaminated by bacteria originating in the saliva during post preparation or through a leaking temporary filling [1]. Disinfection of the prepared post space with sodium hypochlorite or chlorhexidine and antibacterial dressing is suggested [1] (see Figure 2). However, one should bear in mind that the combination of sodium hypochlorite and chlorhexidine results in the formation of a para-chloroaniline (PCA) precipitate [85]. The PCA precipitate could stain dentin and impair the sealing ability of root canal sealers and post and core luting agents by occluding the dentinal tubules [86]. The antibacterial dressing may be Ca(OH)2 combination with CMPC which can reduce the number of cultivable bacteria in the canal [87] or chlorhexidine gel [88, 89] which will not alter the anatomy of the post space when removed. Although according to recent data based on an in vitro study, the mixture of 0.12% chlorhexidine with calcium hydroxide results in an immediate degradation of the CHX [90].

A minimum of 3 mm of residual gutta-percha should be left in the canals after post space preparation [1]. After removal of the gutta-percha, an additional barrier is suggested to prevent coronal microleakage. 2-mm of Triage intraorifice barrier can be suggested [80]; alternatively, 1 mm of Vitrebond placed over the remaining gutta-percha could reduce the risk of recontamination of the apical gutta-percha [84].

Based on the rate of bacterial [10, 49, 50], and endotoxin (5) penetrations, obturated canals which have been exposed to the oral environment for 2-3 months or longer need endodontic retreatment [1]. The decision making process should be further supported by host characteristics, such as oral hygiene, periodontal health, and the position of the tooth in the arch. A ferrule of 1-2 mm of tooth tissue coronal to the finish line of the crown significantly improves the fracture resistance of the tooth and is more important than the type of the material the core and post are made of. The clinician should weigh the advantage of a longer post which provides better retention for the core [47] against the irretrievability of a treatment in case of the development of a periradicular lesion and a need to retreat the tooth endodontically.
